# Deciphering the Role of Macrophages in RSV Infection and Disease

**DOI:** 10.3390/v17101351

**Published:** 2025-10-08

**Authors:** Sara Van Looy, Axelle Fransen, Lotte Jacobs, Sofie Schaerlaekens, Martina Ceconi, Francisco I. Serrano-Cano, Noor Ul Hudda, Laurence Van Moll, Marie De Smedt, Paul Cos, Peter L. Delputte

**Affiliations:** 1 Laboratory for Microbiology, Parasitology, and Hygiene, University of Antwerp, 2610 Antwerp, Belgium; sara.vanlooy@uantwerpen.be (S.V.L.); axelle.fransen@uantwerpen.be (A.F.); lotte.jacobs@uantwerpen.be (L.J.); sofie.schaerlaekens@uantwerpen.be (S.S.); martina.ceconi@uantwerpen.be (M.C.); franciscoivan.serranocano@uantwerpen.be (F.I.S.-C.); noor.ulhudda@uantwerpen.be (N.U.H.); laurence.vanmoll@uantwerpen.be (L.V.M.); paul.cos@uantwerpen.be (P.C.); 2 Infla-Med, University of Antwerp, 2610 Antwerp, Belgium

**Keywords:** macrophages, respiratory syncytial virus, innate immunity, pulmonary infection, cytokines, phenotype plasticity

## Abstract

Respiratory syncytial virus (RSV) is a major cause of severe respiratory infections in infants, the elderly, and immunocompromised persons. Innate immune responses to RSV, which are crucial for containment of the infection yet may also be linked to severe disease, are well-studied in the main RSV target cells, respiratory epithelial cells, but the role of pulmonary macrophages (MΦs), key innate immune regulators, remains incompletely defined. This review addresses the interaction of RSV with MΦ, discussing the susceptibility of these cells to productive infection, and MΦ responses to RSV, including cytokine and chemokine release and inflammasome activation. Furthermore, factors contributing to variability in MΦ infectivity and responses, such as MΦ polarization, age, differences in RSV isolates, co-infections, and prior innate priming, are presented. Finally, the review highlights discrepancies observed across experimental models, MΦ origins, and RSV isolates used, complicating the interpretation of MΦ-RSV interactions, thereby underscoring the need for standardized methodologies.

## 1. Introduction

Human RSV was discovered more than 60 years ago and is classified in the *Pneumoviridae* family, genus *Orthopneumovirus* [1]. RSV is a medium-sized (120–300 nm diameter) pleomorphic enveloped virus with a non-segmented, negative sense, single-stranded RNA genome. The ~15.2 kb genome is composed of 10 genes that encode for 11 proteins, of which three are incorporated in the viral envelope: the attachment (G) glycoprotein, the fusion (F) glycoprotein and the small hydrophobic (SH) protein [2]. The G protein is involved in host cell attachment, and the F protein is responsible for cell entry and spread. Meanwhile, the SH protein, the smallest and least abundant protein on the viral envelope, is not required for viral entry but shows viroporin activity [3]. The RSV particle comprises five other structural proteins, the large (L) protein, nucleocapsid (N) protein, phosphoprotein (P), inner envelope membrane protein (M1), and the M2.1 and M2.2 protein which are encoded from the M2 gene that contains two overlapping open reading frames (ORFs) [4]. In addition, RSV encodes two non-structural proteins, NS1 and NS2, which are the first to be transcribed upon infection. Although not incorporated into the virion, they play a key role in modulating the host immune response [5].

RSV infection is a leading cause of acute respiratory tract infections worldwide. It is responsible for an estimated 3.4 million hospitalizations and 95,000 to 150,000 deaths each year among children under five years of age, with the highest burden observed in low- and middle-income countries (LMICs) [6,7]. Most RSV infections manifest as mild upper respiratory tract infections with common cold-like symptoms such as cough, rhinorrhea, and coryza. However, it can cause a spectrum of illnesses encompassing upper respiratory tract infections (URTI) and lower respiratory tract infections (LRTI), including pneumonia and bronchiolitis [8]. In addition, RSV infection in infants has been associated with the development of asthma, wheezing, and other chronic lung diseases later in life [9]. These clinical outcomes are largely attributed to the host response to RSV, rather than solely by virus-associated cytopathology [10,11]. However, evidence suggests that higher viral load is additionally associated with more severe immunopathology, suggesting that viral replication fitness may contribute in disease severity [12,13]. The burden of RSV-associated disease is disproportionately high in children below five years (particularly infants under six months), as well as in adults aged 65 years and older, and individuals with specific comorbidities. At present, the standard of care for patients with serious RSV illness is limited to supportive care including supplemental oxygen and intravenous fluids. However, effective passive immune prophylaxis is available for children up to 24 months of age and includes palivizumab (Synagis^®^). Due to cost and dosing constraints, prophylaxis with palivizumab for solely very-high-risk infants has recently been replaced by the approved nirsevimab (Beyfortus^®^). This long-acting monoclonal antibody requires only a single intramuscular injection for season-long protection [14,15]. More recently, the first RSV vaccines were approved: Arexvy^®^ [16] of GSK and Abrysvo^®^ of Pfizer, both for adults ≥60 years of age. The latter is also approved for use in pregnant women [17]. In addition, the first mRNA-based RSV vaccine, mRNA-1345 (mRESVIA^®^), was approved for use in adults ≥60 years of age [18,19,20].

Despite the recent advances in RSV prevention options, gaps remain in our knowledge of the drivers of severe respiratory RSV disease. Respiratory epithelial cells play a central role in RSV pathogenesis, not only as primary target cells of infection but also as active participants in initiating immune responses and inflammation. Pulmonary MΦs on the other hand are generally known as key immune cells in the lung, yet the role of these MΦs in RSV disease is less clear. Similarly, our current understanding of the interaction of RSV with MΦs is limited. A better knowledge of the interaction of RSV with MΦs, potentially leading to infection, and how MΦs respond to this encounter, can improve our understanding of RSV infection, immunopathology and disease.

With this review, we will summarize the current knowledge regarding the interaction of RSV with MΦ and discuss the known and suggested roles of MΦ in RSV disease, reflecting the complexity and variation in the literature and critical gaps in our understanding.

## 2. Situating MΦs in the Overall Innate Immune Response Against RSV

**Figure 1:**
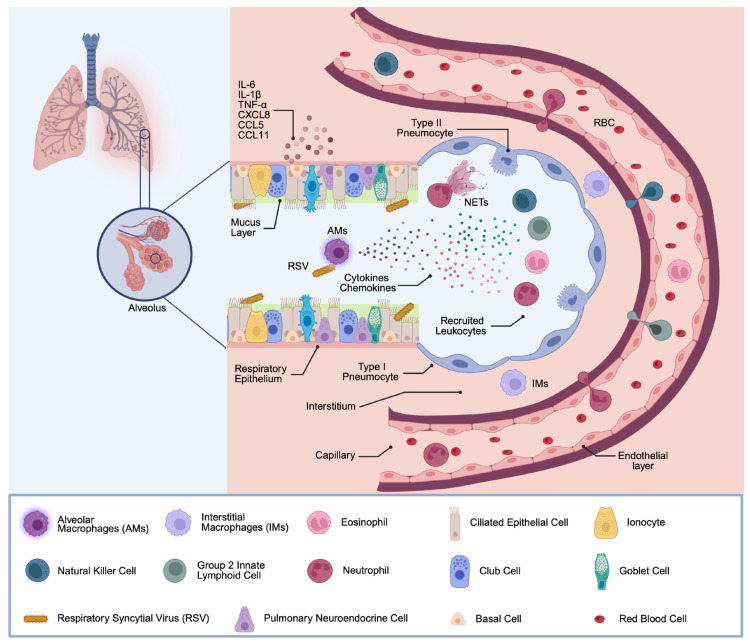
General Innate Immune Response after RSV Infection. The airway epithelium and mucus layer represent the first immune barrier during respiratory infections. The epithelial cells are covered by mucus, contributing to protection from inhaled pathogens. After RSV recognition in the airways by lung epithelial cells and MΦs, these cells secrete inflammatory mediators, such as chemokines and cytokines. This in turn promotes recruitment of NK cells, ILC2s, eosinophils, and neutrophils. These recruited immune cells also release inflammatory cytokines, inducing a second round of inflammatory cytokine secretion at the site of infection. (Created with BioRender software).

The respiratory epithelium serves as a frontline defense against respiratory infections by functioning both as a physical barrier that blocks foreign material from reaching underlying tissues and as an immunological barrier that initiates a protective inflammatory response when stimulated [21]. These epithelial cells are however also the primary target for RSV infection within the airways (Figure 1) [22]. After passing the mucus layer that covers the epithelial cells, RSV initially binds to physiological receptors such as CXCR3, heparin and nucleolin [23]. In addition, it can be recognized by different pattern recognition receptors (PRRs), including Toll-like receptors (TLRs), retinoic acid-inducible gene-I-(RIG-I-) like receptors (RLRs), and nucleotide-binding oligomerization domain-(NOD-) like receptors (NLRs) [24,25]. In response, the synthesis of various chemokines and cytokines, such as interleukin 6 (IL-6), IL-1β, TNF-α, CXCL8, CCL5, and CCL11 [26,27], is initiated. These mediators establish an antiviral state and drive the recruitment of additional immune cells to the site of infection.

Upon RSV infection, several immune cells, usually absent from healthy lungs, rapidly infiltrate the infected tissue in large numbers. Neutrophils account for approximately 80% of infiltrated cells and contribute to pathogen clearance via phagocytosis, the release of neutrophilic granule content (e.g., elastase and myeloperoxidase), and the formation of neutrophil extracellular traps (NETs) [4,28,29]. Besides neutrophils, natural killer (NK) cells are attracted and activated in the early phase of infection and mediate cytotoxic killing of infected cells, as well as the secretion of IFN-γ, which promotes Th1-type immunity [30]. Group 2 innate lymphoid cells (ILC2s) are also expanded in the RSV-infected lung and secrete type 2 cytokines, including IL-5 and IL-13, which promote eosinophil recruitment and mucus hyperproduction [31]. Eosinophils are a minority cell population of the luminal infiltrate during RSV infection; nevertheless, they contribute via phagocytic activity and degranulation, defined by the release of eosinophil cationic protein (ECP) and eosinophil-derived neurotoxin (EDN) [32].

Resident lung MΦs occupy a niche in the respiratory immune response. Two clearly distinct subsets of lung-resident MΦs have been characterized, namely, alveolar macrophages (AMs) and interstitial macrophages (IMs). AMs, residing on the luminal surface of the alveoli, account for over 95% of the leukocytes in the airways in steady-state conditions [33]. Owing to their direct contact with the external environment at the alveolar–capillary membrane, they are among the first immune cells to encounter RSV [34]. They are instrumental in phagocytosing viral particles and debris, and the orchestration of the immune response to infection, by producing a broad array of cytokines and chemokines that in turn influence the recruitment and activation of other immune cells. AMs also play a role in the modulation of epithelial barrier function [8,35]. Of interest, AMs are known for their immunoregulatory capacity; under homeostatic conditions, they promote tolerance to inhaled antigens, but during infection, their phenotype can shift towards a more inflammatory state, contributing to both viral control and, if not well controlled, tissue injury [36,37]. In contrast, IMs are typically located within the lung parenchyma. IMs are less well characterized in the context of RSV infection, yet some evidence suggests that their cytokine response, including IFN and TNF, contributes less to a pro-inflammatory status in RSV infection [38]. Collectively, lung MΦs orchestrate the recruitment and activation of innate immune cells in the RSV-infected lung and must tightly regulate a balanced cascade of signaling events with both pro- and anti-inflammatory characteristics to facilitate virus clearance followed by resolution of inflammation and return to steady-state conditions.

RSV, however, like other viruses, has evolved multiple mechanisms to modify host immune responses to allow virus replication [39,40]. The balance and timing of early innate responses are major determinants of the outcome of RSV infection: delayed, insufficient, or dysregulated responses can result in inefficient viral clearance, allowing high levels of RSV replication and spread towards the lower respiratory tract, thereby enhancing disease severity [9,41]. For RSV, several innate immune components have already been implicated in this dysregulation. For instance, elevated levels of the neutrophil chemoattractant CXCL8 have been associated with increased disease severity [42]. Neutrophilic inflammation has further been linked to impaired early mucosal responses [43,44]. In addition, the dual role of NK cells in RSV defense, providing protective cytotoxicity while potentially suppressing antibody responses, illustrates the delicate balance between protective and pathogenic immune activities [30]. Despite the central role of MΦs as major responders in the respiratory system, and as key initiators of the innate immune cascade, knowledge on their interaction with RSV remains incomplete [45].

## 3. RSV Infection of MΦs: Absent, Abortive or Productive?

Airway epithelial cells are the primary target for RSV infection; however, viral RNA and proteins have also been detected in AMs from naturally infected individuals [46,47]. This suggests that RSV may replicate in MΦs or may exploit these as vessels for virus dissemination, or even long-term persistence, as previously shown for Influenza and COVID-19 [48]. Alternatively, the presence of viral RNA or proteins in MΦ might result from phagocytosis of RSV particles or uptake of apoptotic bodies from infected epithelial cells, rather than productive infection [49]. The outcome of RSV interaction with MΦs remain inconsistent across studies, ranging from productive infection to abortive infection, where some viral proteins may be expressed without production of infectious virions [50,51,52]. Some studies also suggest persistent MΦ infection, with a slow but steady level of continuous viral replication [52,53,54]. These widely varying outcomes are likely attributable, at least in part, to the heterogeneity of MΦ in vitro models and the species origin of MΦs employed in these studies (Table 1) [55].

Human MΦs display considerable heterogeneity and undergo continuous remodeling throughout life, a complexity that is not faithfully reflected in murine AMs [62]. In addition, many alternative MΦ models commonly employed in experimental studies fail to capture the tissue-specific imprinting provided by the pulmonary environment, raising uncertainty as to whether they accurately mirror the biological phenotype of lung-resident MΦs. In this regard, studies using primary MΦs isolated from bronchoalveolar lavage fluid (BALF) represent the most physiologically relevant approach. Yet, this research was conducted in the 1990s [61], and no recent investigations of RSV infection in BALF-derived primary MΦs have been reported. The limited use of this model is largely attributable to ethical and methodological challenges, as MΦs must be freshly isolated for each experiment and cannot be propagated in long-term culture [63]. By contrast, bone marrow–derived MΦs (BMDMs), although frequently used as surrogates, fail to accurately recapitulate MΦ immune responses observed during primary lung infection or injury. BMDMs are considered to differ substantially from AMs in their developmental origin (monocyte-derived versus embryonically seeded), immune responsiveness, metabolic activity, proliferative capacity, and tissue repair functions, underscoring the limitations of extrapolating findings from BMDMs to lung-resident MΦs [64]. Furthermore, to allow meaningful comparison across studies, the viral strain or isolate used must also be considered, as this can substantially influence the outcome of MΦ infection. For influenza A virus, for instance, AM infection rates vary markedly depending on the strain: highly pathogenic avian influenza virus (HPAIV) H5N1 productively infects up to 84% of MΦs, whereas seasonal H3N2 and H1N1 strains typically result in much lower, largely unproductive infection rates of around 7% [65]. Similarly, for coronaviruses, both SARS-CoV and HCoV-229E can productively infect MΦs, although the replication for SARS-CoV appears to be abortive [66]. An RSV study reported divergent outcomes in MΦ infection depending on the virus isolate used [55]; however, the underlying mechanisms explaining these isolate-dependent differences have not yet been systematically investigated. These observations highlight that variation in viral strain, alongside differences in MΦ subtype and experimental model, critically shapes infection outcomes. As a result, it remains challenging to draw firm conclusions regarding RSV infection and replication in MΦs and its contribution to disease pathogenesis. Nevertheless, RSV infection, either productive or abortive, remains plausible and may alter MΦ functions in ways that exacerbate disease severity, analogous to influenza virus infection, where viral replication within MΦs can drive excessive proinflammatory cytokine production [67,68].

## 4. MΦ Responses to RSV Exposure

**Figure 2:**
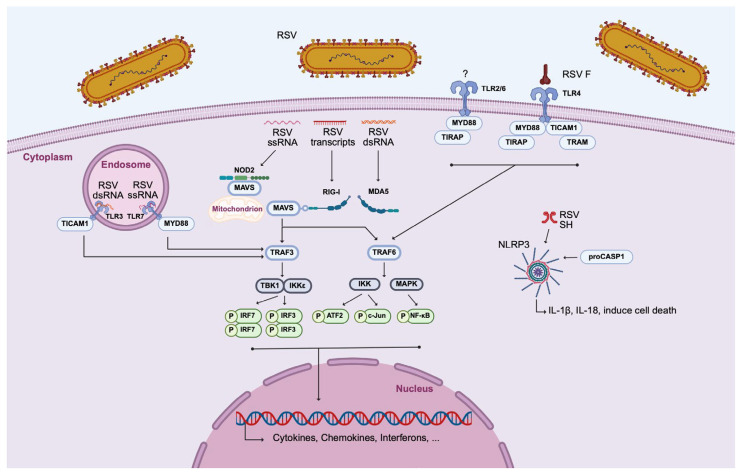
MΦ activation by cell–RSV interaction. Three classes of PRRs are involved in the recognition of RSV by MΦs: TLRs, RLRs, and NLRs. ATF2, activating transcription factor 2; IKK, inhibitor of nuclear factor kappa-B kinase; IRF3, interferon regulatory factor 3; IRF7, interferon regulatory factor 7; MAPK, mitogen-activated protein kinase; MAVS, mitochondrial antiviral-signaling protein; MDA5, melanoma differentiation-associated protein 5; MyD88, myeloid differentiation primary response protein MyD88; NF-κB, nuclear factor-kappa B; NLR, nucleotide-binding oligomerization domain (NOD)-like receptors; NLRP3, NOD-like receptor family, pryin domain containing; NOD2, nucleotide binding oligomerization domain-containing protein 2; RIG-I, retinoic-acid-inducible gene-I; RLR, RIG-I-like receptor; RSV, respiratory syncytial virus; TBK1, tank binding kinase 1; TICAM1, toll/interleukin-1 receptor domain-containing adapter molecule 1; TIRAP, toll/interleukin-1 receptor domain-containing adapter protein; TLR, Toll-like receptor; TRAF3, tumor necrosis factor receptor-associated factor 3; TRAF6, tumor necrosis factor receptor-associated factor 6; and TRAM, Toll-like receptor adaptor molecule. (Created with BioRender software).

MΦs are pivotal orchestrators of the innate immune response against RSV infection, where their ability to detect viral components through PRRs triggers a rapid and robust antiviral response. This early recognition enables MΦs to initiate a cascade of signaling pathways and the production of a broad array of inflammatory mediators, including interferons, cytokines and chemokines (Figure 2) [33,69]. PRRs are the primary sensors responsible for detecting distinct molecular signatures, such as viral RNA or proteins. To further illustrate these processes, the PRRs that predominate the antiviral immune response against RSV infection are described. Three major PRR classes are implicated in RSV recognition by MΦs: RLRs, NLRs, and TLRs. Considering the RLRs, RIG-I and melanoma differentiation-associated protein 5 (MDA5) bind RSV transcripts and dsRNA in the cytosol, respectively. Upon ligand binding, both receptors signal through the downstream mitochondrial antiviral-signaling protein (MAVS), which in turn recruits adaptor molecules TRAF3 and TRAF6. TRAF3 activates the two kinases TBK1 and IKKε, which phosphorylate the transcription factors IRF3 and IRF7, leading to activation of type I interferon gene expression. Simultaneously, TRAF6 activates the MAPK and the IKK kinase complexes, resulting in the phosphorylation of ATF2/c-Jun and NF-κB. These transcription factors then translocate to the nucleus, inducing the transcription of cytokines and chemokines, such as interferons and RANTES [39,70,71,72].

Among the NLRs, NOD2 recognizes ssRNA of RSV and follows a similar downstream pathway as the RLRs. Furthermore, the NLRP3 inflammasome, a multiprotein complex composed of NLRP3, adaptor protein (ASC), and pro-caspase-1, is activated by the RSV SH protein. Once assembled, the inflammasome facilitates caspase-1 activation, leading to the processing and secretion of proinflammatory cytokines IL-1β and IL-18, as well as the induction of pyroptotic cell death [70,73]. TLRs, located on the cell surface or within endosomal compartments, play a critical role in RSV detection by initiating signaling cascades through the recruitment of specific adaptor proteins. Although the precise mechanism by which the TLR2/6 heterodimer detects RSV virions in AMs remains unclear, its activation has been shown to recruit the adaptor proteins MyD88 and TIRAP, leading to NF-κB-dependent production of cytokines and chemokines [39,70,71,72]. Furthermore, RSV infection also induces the expression of TLR3 in MΦs, enabling the recognition of viral dsRNA within endosomes [74]. This receptor signals exclusively through TICAM1, leading to downstream activation of antiviral pathways. TLR7, which detects ssRNA and signals via MyD88, has been shown by Gantier et al. to respond to RSV in human THP-1 cells [75]. The involvement of TLR4 in RSV recognition remains controversial. Although it has been proposed that the RSV F glycoproteins activate TLR4, the biological relevance of this interaction is not yet fully understood [39,70]. TLR4 is unique in the fact that it can signal through both MyD88/TIRAP and TRAM/TICAM1. All those adaptor molecules ultimately converge on TRAF3 or TRAF6, triggering transcriptional programs that result in the expression of antiviral cytokines and effector molecules [39,70].

Although AMs are recognized as early producers of inflammatory mediators during RSV infection, only few studies have characterized their specific contributions to cytokine and chemokine production or their precise role in viral control (Table 2). Notably, Goritzka et al. demonstrated that, in murine models, AMs represent the predominant source of type I interferons following RSV infection, surpassing contributions from epithelial cells and DCs [33]. Nevertheless, a range of key mediators commonly detected during the early stages of infection, including IL-1β, IL-2, IL-5, CXCL1, and CCL2, are not produced by MΦs, underscoring the need to delineate their cell-type-specific sources [76]. Understanding the MΦ-specific contributions to cytokine and chemokine production is of particular importance given the established links between inflammatory mediator profiles and RSV disease severity and prognosis. For example, Bermejo-Martin et al. reported persistently elevated levels of IL-6, IL-1Ra, IL-1β, G-CSF, IL-8, MCP-1, MIP-1α, and MIP-1β in RSV-infected patients across multiple clinical time points compared to healthy controls [77]. In line with these findings, Vázquez et al. proposed that cytokine expression signatures in the respiratory tract could serve as prognostic biomarkers for disease severity [78].

Interestingly, as shown in Table 2, MΦs can produce IL-4, IL-13, and IL-10 in response to RSV exposure. This finding is somewhat paradoxical, since these cytokines are traditionally classified as anti-inflammatory mediators that suppress early proinflammatory responses. The exact nature of this remains elusive, but one could speculate that this production in the context of RSV infection may reflect a regulatory attempt to limit tissue damage and restore alveolar homeostasis. Such a mechanism was also suggested for IL-10 in RSV-infected human AMs, where its induction appears to counterbalance exuberant proinflammatory responses and which may help maintain tissue integrity [85,90,99]. This duality underscores the remarkable plasticity of MΦ responses, whereby the same cell population can contribute both to antiviral defense and to resolution of inflammation. Comparable phenomena have been observed in other respiratory viral infections, such as influenza A virus, where infected MΦs simultaneously produce antiviral interferons and regulatory cytokines, suggesting that this capacity to mount mixed cytokine responses may represent a conserved strategy among respiratory viruses to shape host immunity while sustaining infection [68].

Table 2 further illustrates that nearly all MΦ studies have relied on a small set of long-established laboratory strains, most notably RSV A2 and Long. These prototypic strains have been invaluable for elucidating the fundamental virology of RSV and the functions of its proteins; however, accumulating evidence indicates that they can differ considerably from other laboratory and contemporary clinical strains in cytopathology, antigenicity, and pathogenicity, both in vitro and in vivo [100,101]. Contemporary RSV isolates show natural variation. Clinical isolates are first classified as RSV-A or RSV-B and further subdivided into genotypes that co-circulate and shift in dominance between seasons and geographic regions. Since 2014, ON1 and BA lineages have predominated among RSV-A and RSV-B isolates, respectively. ON1 viruses harbor a 72-nucleotide duplication in the C-terminal region of the G glycoprotein that adds potential O-glycosylation sites, whereas BA viruses possess a 60-nucleotide duplication in a hypervariable region of the RSV-B G gene [102,103]. Comparative genomic analyses, such as those by Stobbelaar et al., highlight that RSV-A displays greater genetic diversity than RSV-B [100]. Importantly, epithelial cell studies already demonstrate that such viral variation influences cytokine production. Villenave et al. reported marked differences in cytokine secretion between A2 and recent pediatric clinical isolates in primary bronchial epithelial cells (PBECs) [104], Stokes et al. observed genotype-dependent variation in IL-13 production in murine lungs [105], and Levitz et al. described differential IL-6 induction between RSV-B isolates [106]. By contrast, MΦ data remain almost entirely derived from the A2 and Long strains, leaving open the question of how currently circulating viruses modulate MΦ cytokine responses. To address this gap, future MΦ studies should systematically incorporate recent clinical isolates.

Taken together, PRR engagement and downstream signaling provide an essential framework for MΦ activation during RSV infection, but the breadth of cytokines produced, ranging from strongly proinflammatory to potently anti-inflammatory mediators, highlights the complexity of their functional programming. This complexity may in part explain the divergent outcomes observed across studies and underlines the importance of contextual factors—such as viral strain, infection history, and MΦ phenotype—in shaping MΦ responses.

### 4.1. Effect of MΦ Phenotype

MΦs represent a functionally diverse population, consisting of a wide and dynamic spectrum of activation states. At the polar ends of this spectrum lie what can be called the classically activated M1 phenotype, which is predominantly pro-inflammatory, and the alternatively activated M2 phenotype, associated with anti-inflammatory responses, tissue repair, and fibrosis [98,107]. Both activation states are considered essential during the course of viral infection. M1 MΦs conduct the initial inflammatory response, via increased phagocytic activity and production of pro-inflammatory cytokines and large amounts of reactive oxygen and nitrogen species. Inversely, M2 MΦs are necessary for the repair of damaged tissue in the aftermath of the viral infection via production of IL-10, IL1Rα, IL-4, IL-3, and TGF-β [98,107,108]. Important to note is that the M1/M2 is a simplified conceptual framework and does not capture the full phenotypic diversity of MΦ activation in vivo. MΦs can shift between intermediate or hybrid states and between phenotypes in response to environmental cues [66]. Both intrinsic factors, such as ontogeny, and extrinsic signals from the tissue microenvironment, particularly the alveolar niche [109], influence MΦ polarization, with strong evidence that the lung environment plays a dominant role in shaping AM identity, shown in murine models [110,111]. Recent work by Kudlik et al. demonstrated the capacity for M1 MΦ to repolarize toward alternative phenotypes depending on cytokine context: reduced IL-10 and TNF-α levels with increased Ym1 expression promoted a shift toward an M2a-like state, whereas increased IL-10 and reduced TNF-α induced a shift toward the M2b phenotype [112]. These findings highlight the remarkable plasticity of MΦs and the context-dependent nature of their functional programming.

During RSV infection, the extrinsic MΦ phenotype determination may be driven through three possible pathways: (i) direct virus–MΦ interactions, (ii) cytokine signaling, and (iii) intracellular communication with epithelial and immune cells. For instance, RSV-induced IFN-γ production by NK cells and CD8^+^ T cells is known to skew MΦs to a proinflammatory phenotype (M1), enhancing antimicrobial responses through induction of NADPH oxidase and inducible nitric oxide synthase (iNOS) [113,114,115,116]. IFN-γ also upregulates MHC class II expression, thereby augmenting the antigen presenting capacity of MΦs [115]. RSV-infected airway epithelial cells can also contribute to M1 polarization through intercellular signaling via the Notch–Jagged pathway or the ligand–receptor interactions involving CD200 and program death-ligand-1 [36,117].

The phenotypic status of MΦs has direct implications for both viral control and pathogenesis. M2-polarized MΦs have been shown to be more permissive to productive RSV replication and can enhance viral infection of epithelial cells in co-culture systems. In contrast, M1 MΦs limit viral spread, suggesting a protective role during infection [118,119]. These findings emphasize the importance of MΦ priming in shaping disease outcome. Interestingly, while viral pathogens such as RSV often induce M1-like polarization to promote effective viral clearance [120,121], commensal microbes can modulate this response. For example, *Staphylococcus aureus*, a common component of the airway microbiota, promotes M2 polarization and has been shown to dampen M1-driven immunopathology during influenza infection, highlighting the complexity of host–microbe–virus interactions [122]. Finally, RSV-induced MΦ programming may have long-term consequences. In a study by Didierlaurent et al., primary RSV infection in mice led to persistent functional reprogramming of AMs, which months later showed diminished responses to TLR agonists. This suggests that RSV can induce a sustained anti-inflammatory state that may help limit excessive inflammation during reinfection but could also compromise effective immune responses [123].

### 4.2. Effect of Age-Related Differences

The most severe clinical manifestations of RSV infection are observed at the extremes of age, namely in neonates and the elderly. While both groups are susceptible to severe disease, the mechanisms driving this susceptibility differ. Notably, AMs exhibit age-related changes that may diminish their capacity to mount effective host defenses [124]. In neonates and infants, this vulnerability is linked to multiple elements, such as immunological immaturity and underdeveloped airways. Although the immunological profile of human neonatal lung MΦs remains incompletely characterized, studies in non-human primates, rodents, and rabbits have demonstrated maturational deficiencies in pulmonary MΦ function during early life [125]. For instance, the alveolar milieu is enriched with regulatory cytokines such as IL-10 and TGF-β, which may contribute to a tolerogenic phenotype and dampen antiviral MΦ responses. This cytokine-rich environment could suppress NF-κB and IRF3 signaling, leading to reduced production of type I interferons and impaired viral clearance [126,127]. TGF-β1 has further been identified as a key regulator that skew neonatal antigen-presenting cell responses—potentially extending to MΦs—towards an altered Th1/Th2 balance upon RSV exposure [128]. Beyond cytokine-mediated regulation, intrinsic metabolic and transcriptional programs also critically influence neonatal MΦ responses. For example, in young infants the expression of the metabolic regulator mechanistic target of rapamycin (mTOR)—which supports a shift toward anaerobic metabolism during infection—is reduced compared with adults, contributing to a more tolerogenic functional profile [34]. Ex vivo studies of human cord blood-derived MΦs and neonatal mouse models reveal elevated expression of the immunoregulatory cytokine IL-27, consistent with an immunosuppressive phenotype [34,129]. In the setting of RSV infection, MΦ-T cell interactions are also developmentally regulated. Specifically, infant MΦs exhibit delayed secretion of IFN-γ upon interaction with CD4^+^ T cells, which biases their polarization toward an M2-like phenotype. This shift compromises their antiviral capacity, resulting in reduced phagocytosis of viral particles and impaired recruitment of additional immune effector cells, such as T lymphocytes [130].

In contrast, disease severity in the elderly is frequently linked to age-related decline in immune function, a process termed immunosenescence. This state is characterized by “inflammaging,” encompassing diminished adaptive immunity, reduced T-cell repertoire diversity, and chronic low-grade inflammation. In MΦs, oxidative stress, mitochondrial dysfunction, and dysregulation of key signaling pathways, including NF-κB and mTOR, further exacerbate age-related immune decline [131]. Within the lung, aging profoundly affects AMs [132]. In vitro analysis of AMs from aged individuals reveal multiple functional impairments compared with those from healthy adults, including reduced oxidative burst, diminished phagocytic capacity, impaired chemokine production, and decreased cell numbers, thereby weakening a critical first line defense [133]. Notably, dysregulated MΦ responses have already been implicated in poor outcomes of other respiratory viruses; for example, aberrant AM activity has been associated with increased influenza-related mortality in older populations. At the molecular level, aging reshapes the AM transcriptome by inhibiting cell cycle pathways. In addition, aging diminishes the capacity of AMs to engulf dying neutrophils, reduces the presence of scavenger receptors such as CD204, and causes the accumulation of neutrophiles during viral infection [124]. In murine models, aging is accompanied by a gradual replacement of embryonically seeded AMs with bone marrow-derived AMs, a process that correlates with heightened inflammatory phenotypes and increased influenza severity [124]. These age-dependent differences in MΦ function highlight the importance of developmental context in shaping innate immune responses to RSV and may provide insight into the mechanisms underlying age-associated disparities in disease severity.

## 5. Conclusions

Despite decades of RSV research, the precise role of MΦs in disease pathogenesis remains a black box. As central sentinels of the innate immune response, MΦs are involved in both innate and adaptive antiviral immunity through a coordinated program of phagocytosis, cytokine production, and antigen presentation. However, their function during RSV infection is highly context-dependent and influenced by variables such as age, ontogeny, tissue microenvironment, infection history, and viral strain.

Multiple studies suggest that MΦs are not merely passive bystanders but actively respond to RSV and can even be infected. Yet, the extent of infection, ranging from absent to abortive to productive, seems to vary depending on the MΦ subtypes, species origin, and RSV strains and isolates used. This heterogeneity complicates interpretation of MΦ–RSV interactions and their downstream consequences. MΦ phenotype is a crucial factor in modulating infection outcomes. M1-polarized MΦs exhibit strong antiviral properties, including enhanced cytokine production, phagocytic activity, and antigen presentation, which contribute to viral control. In contrast, M2-polarized MΦs, associated with tissue repair and immunomodulation, have been shown to be more permissive to RSV replication and may facilitate viral persistence or immune evasion. Notably, MΦs demonstrate remarkable plasticity, with cytokine environments capable of inducing shifts in phenotypes. This highlights the dynamic responsiveness of MΦs to fluctuating microenvironmental cues during infection.

Age-related differences in MΦ biology further influence disease susceptibility. In neonates and older adults, AMs show impaired phagocytosis, diminished chemokine production, and delayed IFN-γ responses, leading to ineffective viral clearance and skewing toward anti-inflammatory phenotypes. Moreover, RSV infection may result in long-term reprogramming of MΦ responsiveness, as evidenced by sustained hypo-responsiveness to TLR ligands in AMs months after infection. This phenomenon underscores the potential for RSV to induce persistent alterations in innate immune function, which may have implications for reinfection susceptibility and vaccine efficacy.

Crucially, MΦ-derived inflammatory mediators play dual roles in host defense and immunopathology. As summarized in Table 2, MΦs release a broad spectrum of cytokines and chemokines, including IFNs, TNF-α, IL-6, IL-10, IL-33, and CCL5, in a temporally regulated manner and with early evidence indicating potential strain-related differences. These mediators coordinate immune cell recruitment, shape adaptive responses, and influence the severity and chronicity of RSV disease. Yet, significant challenges remain in the reproducibility and translatability of experimental findings. Differences in RSV strains and the choice of MΦ model all introduce variability in immune profiles, emphasizing the need to standardize experimental systems and better model physiologic conditions.

Moving forward, several unanswered questions require systematic investigation:

- Do human AMs support productive RSV infection, and if so, does this lead to the release of infectious viral particles?
- To what extent does viral strain or isolate determine RSV replication outcomes in MΦs, and what mechanisms underlie these differences?
- Is the MΦ response during RSV infection protective or deleterious for the host, and how does this vary with age or prior infection history?

Addressing these questions will require integration of human primary MΦs and age-stratified models and use of recently isolated RSV strains. Ultimately, unraveling the multifaceted roles of MΦs in RSV infection is essential to clarify whether they act as viral targets, immune regulators, or both, and to determine how this knowledge might be harnessed for host-directed therapies.

## Figures and Tables

**Table 1:** Overview of infection kinetics outcomes after RSV exposure to murine/human MΦs.

Author	Species	Macrophage Type	Infection Kinetics
Gaona et al. [47] Sarmiento et al. [54] Ruiz et al. [56]	Murine	P388D1	Persistent infection
Miller et al. [52]		MHS	Increase of RSV G protein over time (72 h)
Porto et al. [50] Ravi et al. [57]		PAM	No infection Abortive infection
Sarmiento et al. [54]	Human	U937	Productive infection
Machado et al. [58] Bedient et al. [59]		THP-1	Productive infection
Machado et al. [58] Porto et al. [50]		MDM	Productive infection
Porto et al. [50]		ALM from pluripotent stem cells	No infection
Panuska et al. [60,61]		PAM	Permissive for infection Productive infection

PAM: Primary alveolar MΦs; MDM: monocyte-derived MΦs; ALM: alveolar-like MΦs.

**Table 2:** Inflammatory mediators expressed by MΦs in response to RSV exposure.

Inflammatory Mediator Produced by MΦ	Secretion Time Point (hpi)	Used RSV Strain	Induced Via	Effect During RSV Infection	Model Used
**TGF-β**	2, 4, 8 [79]	RSV A2 [79] Long strain [80]	/	Activates SMAD-2/3 signaling pathway via autocrine action [79] Affects IFN-β production [79] Suppresses iNOS/NO production by MΦs [81]	RAW 264.7 [79] Murine BMDMs [79] MΦ depletion [80]: [24, 48, 72 h increase]
**Type I IFNs** **(IFN-a/IFN-b)**	12 [33,80], 20 [76], 24 [80,82]	RSV A2 [33,76,82] Long strain [80]	MAVS coupled PRR [76]	Interferes with viral replication [79]	Primary murine AMs [33,76,80] MΦ depletion [80,83]: [12, 24 h decrease] Human MDMs [82]
**TNF-a**	6, 16 [84], 20 [76], 24 [80,85]	RSV A2 [76,83,84,85] Long strain [80]	TLR2 and TLR6 [71]	Neutrophil activation and migration to the lungs [86]	Primary human AMs [84] Primary murine AMs [76,80] MΦ depletion [80,83]: [12, 24, 36 h decrease]
**IL-6**	16 [84], 20 [76], 24 [80]	RSV A2 [76,83,84,87] Long strain [80]	TLR4 [87]	Promotion of the differentiation of naïve CD4^+^ and CD8^+^ T cells [78]	Primary human AMs [84] Primary murine AMs [76,80,87] MΦ depletion [80,83]: [12, 24 h decrease]
**IL-33**	72 [88]	RSV A2 [88]	TLR3- and TLR7-MAPK [88,89]	Promotes type 2 responses [78]	RAW 264.7 [88,89]
**IL-4**	24, 48, 72 [90]	Long strain [90]	TLR4 [91]	Polarization towards M2 MΦs [90] Amelioration of lung epithelial damage [90]	Primary murine AMs [90] RAW 264.7 [90]
**IL-13**	24, 48, 72 [90]	Long strain [90]	TLR4 [91]	Polarization towards M2 MΦs [90] Amelioration of lung epithelial damage [90]	Primary murine AMs [90] RAW 264.7 [90]
**IL-10**	24 [85]	RSV A2 [85]	/	Inhibits early cytokine expression through an apparent autocrine mechanism [85]	Primary human AMs [85]
**CXCL10 (IP-10)**	14 [58], 20 [76]	RSV A2 [76] Long strain [58]	/	Promoting leukocyte recruitment and a role in trafficking and functioning of dendritic cells [92]	Primary murine AMs [76] Humans MDM [58]
**CCL3 (MIP-1a)**	20 [76]	RSV A2 [76]	/	Recruitment T- and NK cells [93]	Primary murine AMs [76,80] MΦ depletion [83]: [24 h decrease] MΦ depletion [80]: [24, 48, 72 h increase]
**IL-8 (CXCL8)**	16, 20 [84]	RSV A2 [84]	/	Recruitment of neutrophils [94]	Primary human AMs [84]
**CCL5 (RANTES)**	24 [52,80], 48 [52]	RSV A2 [52] Long strain [80]	/	Recruitment of CD8^+^ T cells [95] Block apoptosis of AMs [95]	Primary murine AMs [80] Murine MHS cells [52] MΦ depletion [80,83]: [24, 36 h decrease]
**CCL4**	12, 24, 36 [80]	Long strain [80]	/	/	Primary murine AMs [80] MΦ depletion [80]: [12, 36 h decrease]
**GM-CSF**	12, 24, 48 [80]	Long strain [80]	/	Block apoptosis of AMs [96] Eosinophil filtration [97] Elongate neutrophil lifespan [98]	Primary murine AMs [80] MΦ depletion [80]: [12, 24, 36, 48 h decrease]

With RAW 264.7, semi-adherent macrophage-like cell line derived from BALB/c mice, transformed by the Abelson leukemia virus; BMDM, bone marrow derived MΦs; iNOS, inducible nitric oxide synthase; MDM, monocyte derived MΦs; MHS, murine alveolar cell line.
